# *Vespula* spp. venom immunotherapy: basophil excitability and Fc_ε_RI/Fc_γ_RIIb expression

**DOI:** 10.1186/s13223-026-01062-w

**Published:** 2026-09-24

**Authors:** Janik Fleissner, Johanna Stoevesandt, Matthias Goebeler, Axel Trautmann, Andreas Kerstan

**Affiliations:** Department of Dermatology, Venereology, and Allergology, University Hospital Würzburg, Josef-Schneider-Str. 2, D-97080 Würzburg, Germany

**Keywords:** Basophil activation test, Hymenoptera venom allergy, Venom immunotherapy, Immune tolerance

## Abstract

**Background:**

Basophils are recognized as important effector cells during the sensitization and effector phases of IgE-mediated allergy. Besides histamine release during anaphylaxis, basophils contribute to early immune polarization as an important source of Th2-associated cytokines. *Vespula* spp. venom immunotherapy (VIT) represents a prime example of clinically highly effective allergen-specific immunotherapy. This study investigated functional and phenotypic changes in circulating basophils during early VIT in a homogenous case series.

**Methods:**

Twenty-four patients eligible for VIT were examined before treatment, after 3 days, and after 1 month of therapy. Basophils were primed with interleukin-(IL-)3 and stimulated with IgE-dependent triggers, including *Vespula* spp. venom and an anti-IgE antibody, as well as the IgE-independent bacterial peptide N-formylmethionyl-leucyl-phenylalanine (fMLP). Basophil activation was assessed by flow cytometric measurement of CD63 expression. In parallel, Fc_ε_RI and Fc_γ_RIIb receptor expression on basophils was quantified, and serum levels of total IgE, *Vespula* spp. venom-specific IgE, and venom-specific IgG_4_ were measured. Changes over time were tested using a mixed-effects model for repeated measures. Paired t-tests, Wilcoxon signed-rank tests, and Friedman tests were used for paired comparisons and non-parametric repeated measures.

**Results:**

After one month of VIT, venom-specific basophil excitability was significantly reduced. In contrast, activation by anti-IgE and fMLP remained largely unchanged. An increase of both Fc_ε_RI and Fc_γ_RIIb expression on basophils was already detectable at day 3 after starting VIT and reached statistical significance at day 28. Serologically, total IgE and *Vespula* spp. venom-specific IgE slightly rose during VIT, but the most pronounced increase was observed for venom-specific IgG_4_.

**Conclusions:**

Data of this study suggest that upregulation of Fc_γ_RIIb together with venom-specific IgG_4_ induction drives venom-specific basophil suppression, possibly by Fc_γ_RIIb-Fc_ε_RI co-aggregation. The overall picture of venom-specific basophil activation, basophil Fc_γ_RIIb expression, and serum IgG_4_ may serve as a biomarker of immune modulation towards tolerance.

**Supplementary Information:**

The online version contains supplementary material available at https://doi.org/10.1186/s13223-026-01062-w.

## Background

Hymenoptera stings are among the most frequent causes of anaphylaxis, and venom allergy represents a prime example of IgE-mediated allergy [1]. Venom allergy in humans provides an opportunity to investigate the effects of allergen-specific immunotherapy in vivo, reflected by changes of cellular and serological parameters in peripheral blood samples. Basophil granulocytes are critically implicated in the pathogenesis of sting-induced anaphylaxis [2].

Basophils are circulating, histamine-containing effector cells [3], which express the high-affinity IgE receptor Fc_ε_RI [4]. They account for less than 1% of peripheral blood leukocytes and originate from common myeloid progenitors in the bone marrow, with interleukin-(IL-)3 promoting their differentiation [5]. Terminally differentiated, mature basophils have a short lifespan of only a few days [6]. Like other leukocytes, they can leave the bloodstream to migrate into tissues via the interaction of homing receptors and adhesion molecules [7]. Basophils carry allergen-specific IgE bound to Fc_ε_RI on their surface, which enables them to rapidly respond to allergens in peripheral blood [8]. Furthermore, basophils contribute to type 2 immune polarization by secreting IL-4 during early immune activation [9]. Thus, they link innate effector mechanisms with IgE-mediated immune responses and contribute to the clinical manifestation of anaphylaxis, particularly in the context of intravascular allergen exposure, such as in *Vespula* spp. venom allergy.

Although *Vespula* spp. venom immunotherapy (VIT) is highly effective in preventing anaphylaxis upon re-sting [1], the mechanisms underlying its clinical efficacy remain incompletely understood [10]. As early as day 5 of ultra-rush VIT, Fc_ε_RI-mediated basophil desensitization has been demonstrated and proposed as an early protective mechanism [11]. Moreover, allergen-specific basophil responses were shown to decrease after completing VIT and correlated with tolerance induction in a long-term sting challenge [12]. While these findings establish that VIT modulates basophil function, phenotypic changes in immunoglobulin receptor expression on basophil surfaces in conjunction with early induction of venom-specific IgG_4_ have received less attention.

In this study, we investigated early functional and phenotypic features of circulating basophils during the first 28 days of VIT in patients with *Vespula* spp. venom allergy in order to explore whether changes in basophil activation and immunoglobulin receptor expression are associated with the early humoral immune response, which may reflect clinical efficacy of VIT. We hypothesized that early VIT selectively reduces allergen-specific basophil activation and that this is accompanied by changes in immunoglobulin receptor expression and venom-specific IgG_4_ induction.

## Methods

### Study design and participants

This prospective single-center study investigated early functional and phenotypic changes in circulating basophils during the first month of VIT. The study was conducted at the Department of Dermatology, Venereology and Allergology, University Hospital Würzburg, Germany. Twenty-four patients with a clear-cut history of Vespula spp*.* sting-induced anaphylaxis eligible for VIT according to the current guideline criteria were studied [1]. Specific sensitization was confirmed by intradermal tests (IDT) with *Vespula *spp. venom and detection of allergen-specific serum IgE levels. Induction of VIT was carried out as previously described [13] using an aqueous allergen product containing a standardized mixture of venoms derived from several closely related *Vespula* spp.(ALK Lyophilisiert SQ™, ALK-Abelló). Informed consent was obtained from all patients, and the study was approved by the Medical Ethics Committee of the Julius-Maximilians-University Würzburg, Germany (reference number 255/13). All clinical investigations were conducted according to the Declaration of Helsinki.

### Basophil activation test

**Fig. 1:**
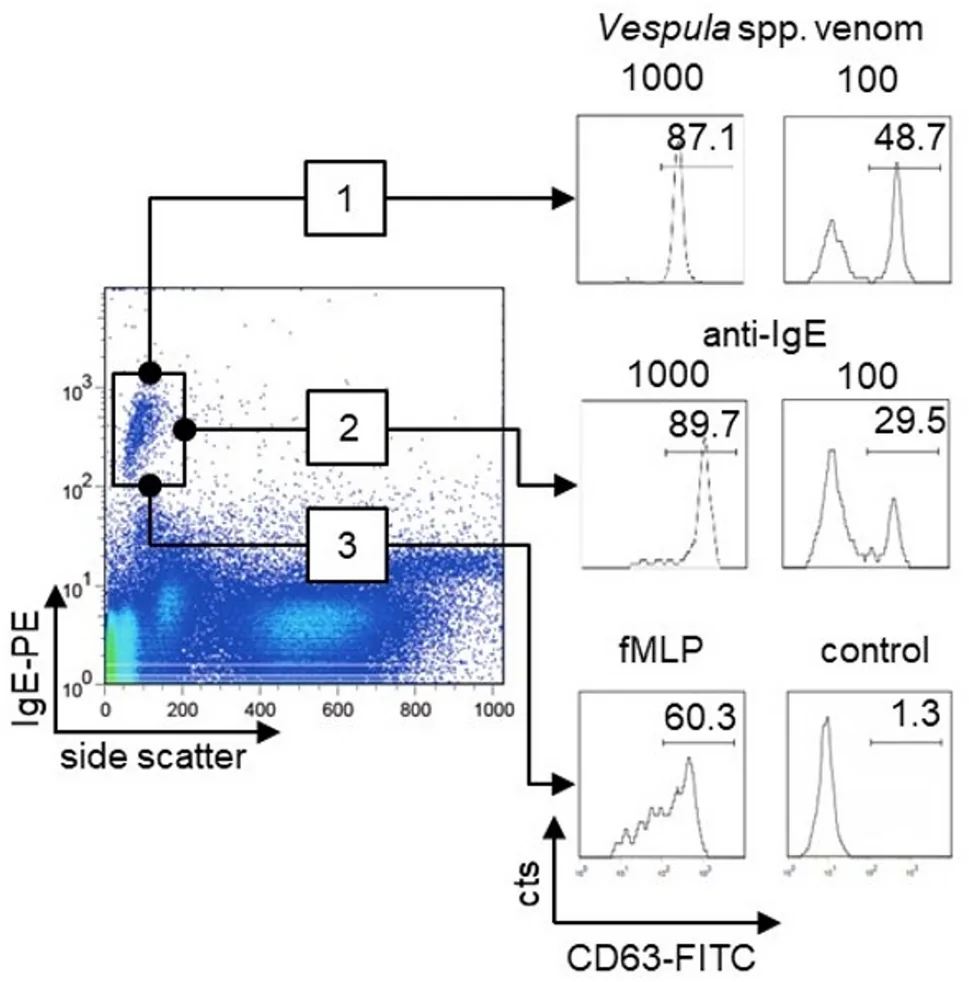
Basophil activation test. Anti-IgE-phycoerythrin-labelled basophils were gated for CD63 expression after stimulation with graded concentrations (ng/mL) of (1) *Vespula* spp. venom, (2) agonistic anti-IgE monoclonal antibody and (3) non-specific stimulation control (fMLP) or negative control. Percentage of CD63 expression is shown. cts, counts.

Basophil activation test (BAT) was performed according to the manufacturer’s instructions (Glycotope Biotechnology, Heidelberg, Germany). Briefly, heparinized whole blood was incubated with 100 or 1000 ng/mL of *Vespula* spp. venom (ALK-Abelló, Hamburg, Germany), or the same concentrations of anti-IgE monoclonal antibody (clone Le27, antibodies-online GmbH, Aachen, Germany) or 2 µM N-formylmethionyl-leucyl-phenylalanine (fMLP, Glycotope GmbH, Berlin, Germany) as a non-specific positive control at 37 °C for 20 min. Degranulation was stopped by chilling samples on ice. Thereafter, samples were stained using anti-CD63-FITC (clone MEM-259, Invitrogen, Thermo Fisher Scientific, Darmstadt, Germany) and anti-IgE-PE (clone MHE-18, BioLegend, San Diego, CA, USA) for 20 min. Finally, whole blood samples were lysed, washed, fixed, and analyzed within 2 h on a FACSCalibur flow cytometer (BD Biosciences, Heidelberg, Germany). Cell acquisition was stopped after record of 1,000 IgE-positive events per sample. Flow cytometry data were analyzed using FlowJo software (version 10). The gating method and exemplary stimulation responses are shown in Fig. 1.

### Receptor counting

Fc_ε_RI and Fc_γ_RIIb (CD32b) expression on basophils was assessed by indirect immunofluorescence using purified mouse anti-human Fc_ε_RI (clone AER-37/CRA1, eBioscience, San Diego, CA, USA) and anti-human Fc_γ_RIIb (clone EP888Y, Abcam, Cambridge, United Kingdom), respectively. Basophil-bound primary antibodies were detected using the FITC-conjugated secondary antibody provided in the QIFIKIT^®^ calibration system (Dako, Glostrup, Denmark). Quantification was performed according to the manufacturer’s instructions using calibration beads and thus converting fluorescence intensity into antibody-binding capacity and receptor numbers per cell.

### Serum immunoglobulin levels

Total IgE, *Vespula* spp. venom-specific IgE, Ves v 5-specific IgE, and venom-specific IgG_4_ were measured by ImmunoCAP (Thermo Fisher Scientific, Darmstadt, Germany) according to the manufacturer’s instructions.

### Statistical analysis

Statistical analysis was performed using GraphPad Prism software (version 8). Data are shown as mean ± standard error of the mean (SEM). To analyze changes over time, a mixed-effects model for repeated measures was used, with time as a fixed effect and individual patient as a random effect. This approach allowed inclusion of patients with incomplete data on day 3 of VIT. Analyses were performed separately for each stimulation condition and concentration. When the overall model was significant, post-hoc pairwise comparisons between time points were performed with Bonferroni correction for multiple testing. For other paired comparisons, including phenotypic basophil analyses and serum immunoglobulin measurements, paired t-tests were used for normally distributed data and Wilcoxon signed-rank tests for non-normally distributed data. Comparisons of fold changes across immunoglobulin classes were analyzed using the Friedman test, followed by post hoc pairwise comparisons as appropriate. Baseline responses to different *Vespula* spp. venom or anti-IgE concentrations were compared within subjects using paired tests. All tests were two-sided, and *p*-values < 0.05 were considered statistically significant.

## Results

### Patient characteristics

The mean age of the 14 male and 10 female patients studied was 44.4 ± 3.0 years. According to the Ring and Messmer anaphylaxis classification [14], one patient experienced a grade I, 14 patients grade II, and nine patients grade III reactions, while no grade IV anaphylaxis was reported. Sensitization to *Vespula* spp. venom was confirmed by both intradermal testing (IDT) and venom-specific IgE measurements. Three patients had concomitant atopy. Patient characteristics are summarized in Table 1. For each patient, basic demographics and diagnostical results (serology, IDT, and BAT at baseline) are shown in Supplementary Table 1.

**Table 1:** Patient characteristics

*Patients*		*n* = 24	
mean age (years)		44.4 ± 3.0	
sex (m/f)		14/10	
*Anaphylaxis grade*		*n* =	
I		1	
II		14	
III		9	
IV		0	
*Threshold of positive IDT µg/mL*		*n* =	
*Vespula* spp. venom			
0.001		2	
0.01		9	
1.0		11	
0.1		2	
Honeybee venom			
0.1		1	
1		4	
*Atopy, n*		3/24	

Age presented as mean ± SEM. IDT, intradermal test; M, male; F, female

### Venom-specific basophil activation decreases during early *Vespula* spp. VIT

To assess excitability of basophils in circulating peripheral blood during early VIT, basophil activation was measured by flow cytometric quantification of CD63 expression after stimulation with *Vespula* spp. venom, agonistic anti-IgE antibody, or N-formylmethionyl-leucyl-phenylalanine (fMLP).

**Fig. 2:**
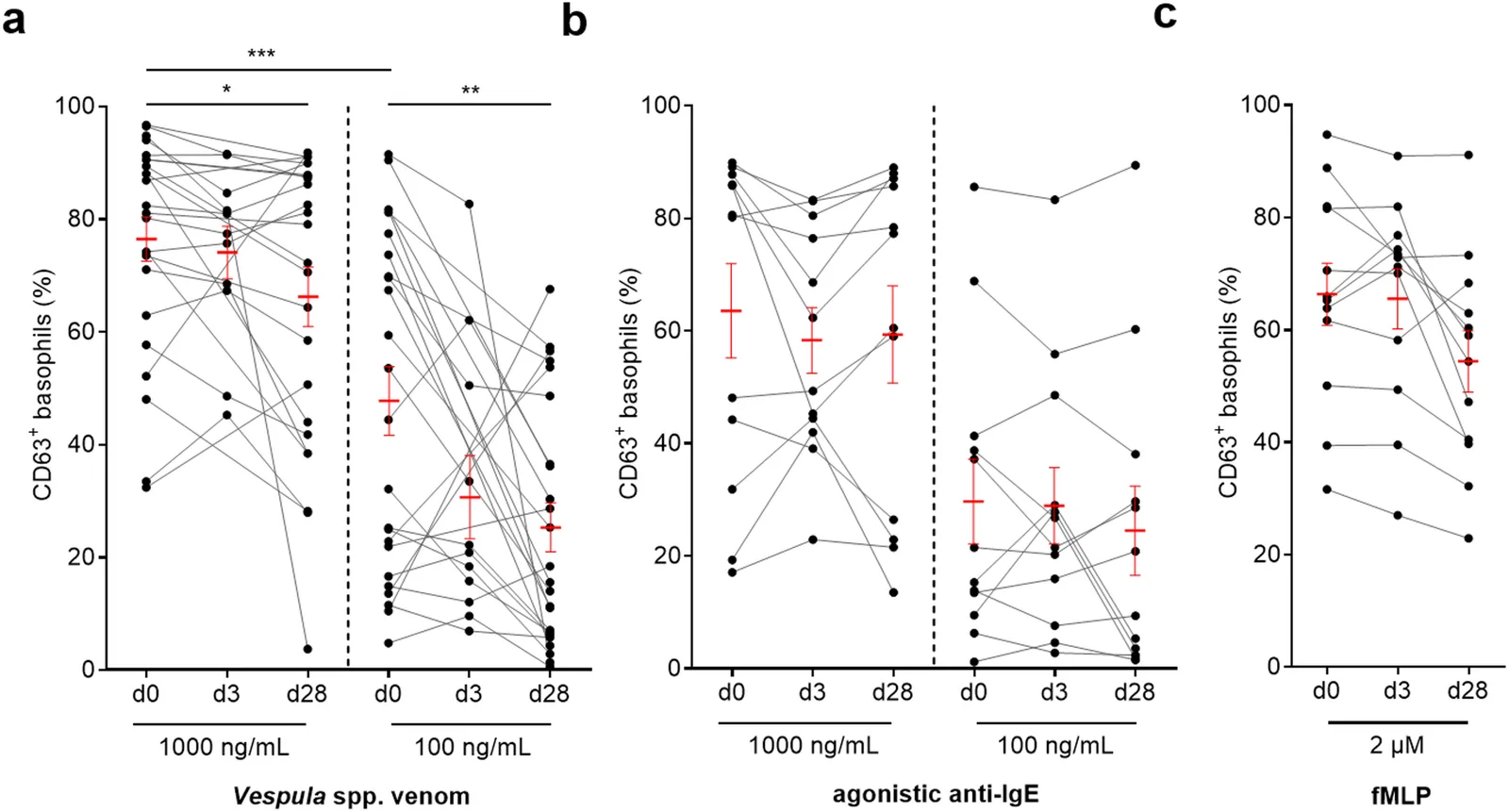
Basophil activation during the course of *Vespula* spp. VIT. **a** Basophils were stimulated with *Vespula* spp. venom, **b** agonistic anti-IgE monoclonal antibody (100 and 1000 ng/mL, respectively) or **c** fMLP as an IgE-independent positive control. CD63 expression was measured by flow cytometry at baseline (d0), day 3 (d3), and day 28 (d28) after initiation of VIT. Data are presented as mean ± SEM. For *Vespula* spp. venom stimulation, *n* = 24 at d0 and d28 and *n* = 11 at d3; for anti-IgE and fMLP stimulation, *n* = 12 at all time points. Statistical analysis was performed using a mixed-effects model for repeated measures followed by post hoc multiple-comparison testing. ns: not significant. *p < 0.05, **p < 0.01, ***p < 0.001.

*Vespula* spp. venom induced dose-dependent basophil activation before starting VIT, with higher CD63 expression after stimulation with 1000 than with 100 ng/mL venom (77.3 ± 3.9% vs. 49.0 ± 6.2%; *p* = 0.0001, Fig. 2a). During the first month of VIT, CD63 expression decreased with both venom concentrations, although the reduction was more pronounced at 100 ng/mL (30.4 ± 7.4% after 3 days, *p* = 0.175, and 23.7 ± 4.3% after 28 days, *p* = 0.002). At 1000 ng/mL, basophil activation decreased to 73.9 ± 4.7% after 3 days (*p* = 0.293) and to 64.9 ± 5.4% after 28 days (*p* = 0.014). Before starting VIT, anti-IgE also induced dose-dependent basophil activation, with higher CD63 expression at 1000 ng/mL than at 100 ng/mL (63.3 ± 8.4% vs. 29.4 ± 7.5%; *p* = 0.001, Fig. 2b). However, anti-IgE-induced basophil activation remained unchanged during the first month of VIT at both concentrations tested. Similarly, fMLP-induced basophil activation remained largely stable during early VIT, although a statistically non-significant reduction was observed at day 28 (Fig. 2c).

### Early VIT increases immunoglobulin receptor expression on basophils

**Fig. 3:**
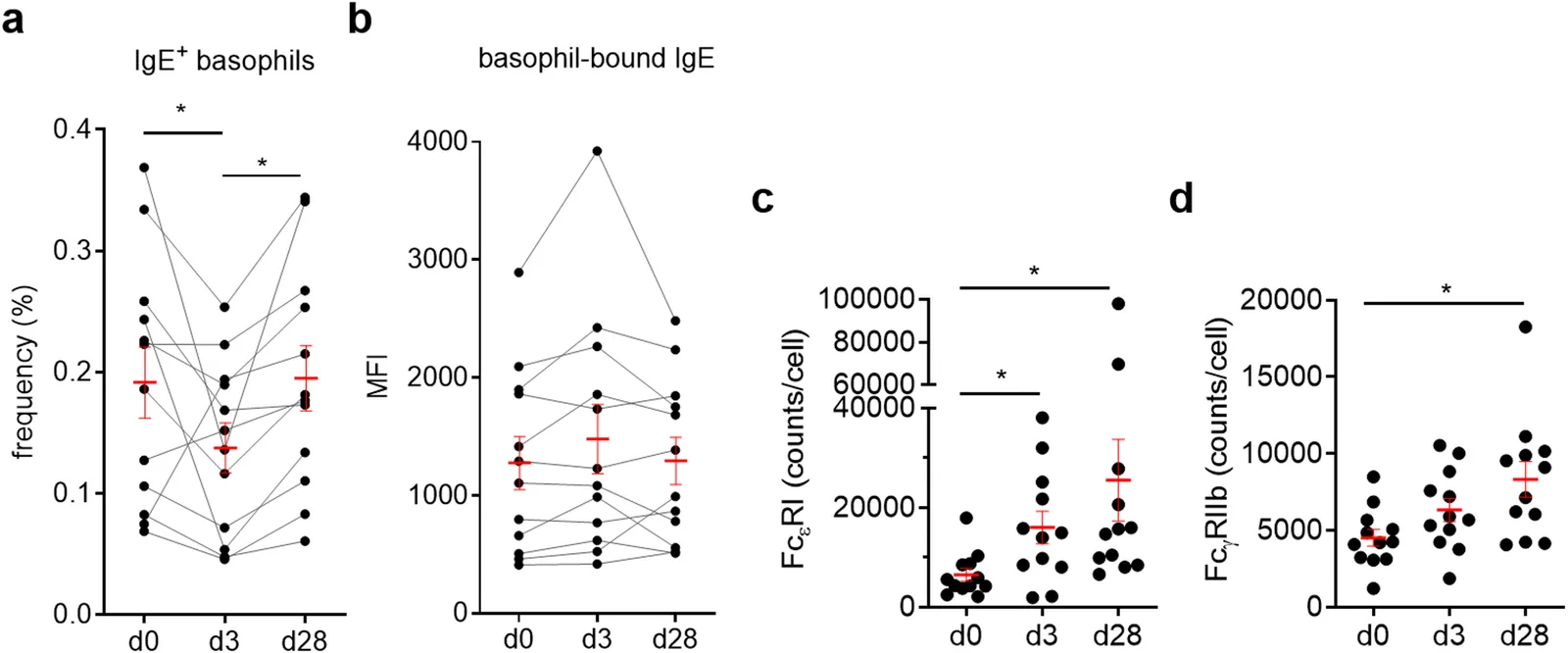
Phenotypic changes in circulating basophils during early *Vespula* spp. VIT. **a** Frequency of circulating basophils, **b** surface-bound IgE on basophils expressed as mean fluorescence intensity, and quantitative receptor numbers of **c** Fc_ε_RI and **d** Fc_γ_RIIb were assessed at baseline (d0), day 3 (d3), and day 28 (d28) of VIT. For panels A and B, measurements were performed in triplicate and averaged for each patient prior to analysis. Data are shown as mean ± SEM; *n* = 12 patients at all time points. *p < 0.05.

We examined whether the observed changes in basophil excitability were paralleled by phenotypic changes. The frequency of IgE^+^ basophils among blood leukocytes showed a transient decrease at day 3 of VIT (0.19 ± 0.03% vs. 0.14 ± 0.02%, *p* = 0.042) and returned to baseline levels by day 28 (0.20 ± 0.03%; Fig. 3a). In contrast, basophil-bound surface IgE levels, quantified as mean fluorescence intensity (MFI), showed no significant change during the observation period (Fig. 3b). Immunoglobulin receptor counting revealed an increase in Fc_ε_RI and Fc_γ_RIIb receptor numbers during VIT (Fig. 3c and d). Fc_ε_RI receptor numbers increased from 6536 ± 1271 per basophil at baseline to 16,013 ± 3269 at day 3 (*p* = 0.012) and 25,493 ± 8231 at day 28 (*p* = 0.041). Fc_γ_RIIb receptor numbers increased from 4518 ± 549 at baseline to 6339 ± 749 at day 3 (*p* = 0.052) and 8331 ± 1159 at day 28 (*p* = 0.013). Thus, the decline in venom-specific basophil activation was accompanied by an increased number of cell surface immunoglobulin receptors.

### Serum immunoglobulin levels during early VIT

**Fig. 4:**
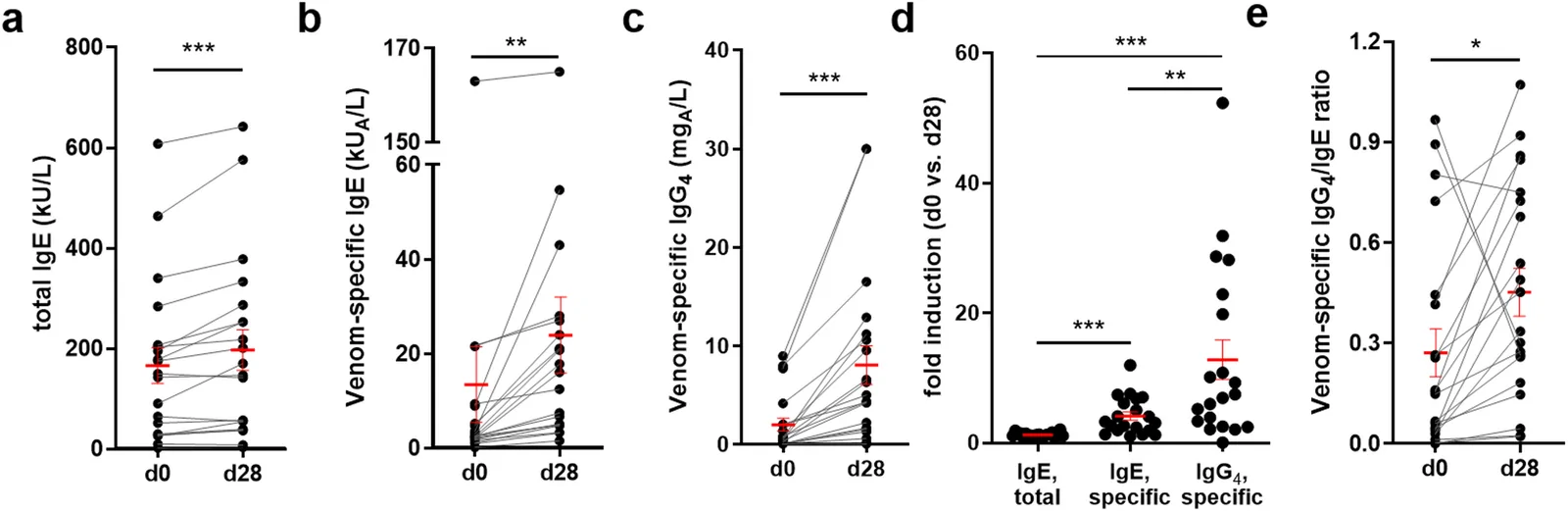
Serum immunoglobulin levels during early *Vespula* spp. VIT. **a** Serum levels of total IgE (kU/L), **b** venom-specific IgE (kU_A_/L), and **c** venom-specific IgG_4_ (mg_A_/L) were measured at baseline (d0) and after 28 days (d28) of VIT. **d** Fold induction from d0 to d28 is shown for total IgE, venom-specific IgE, and venom-specific IgG_4_. **e** Venom-specific IgG_4_/IgE ratio at baseline (d0) and day 28 (d28) in individual patients. Data are shown as mean ± SEM; *n* = 20 patients. Statistical comparisons were performed using paired t tests and Friedman test. *p < 0.05, **p < 0.01, ***p < 0.001.

Serum levels of total IgE, *Vespula* spp. venom-specific IgE, and venom-specific IgG_4_ were measured before and after 28 days of VIT. Total IgE increased from 163.8 ± 35.9 kU/L at baseline to 194.5 ± 40.5 kU/L after 28 days of VIT (*p* < 0.001, Fig. 4a). Of note, we also observed a significant increase in serum levels of venom-specific IgE during this period (13.1 ± 8.0 kU_A_/L to 23.6 ± 8.1 kU_A_/L, *p* = 0.001, Fig. 4b). Consistent with this finding, serum levels of major allergen Ves v 5 specific IgE also increased (8.4 ± 5.6 kU_A_/L to 16.0 ± 6.0 kU_A_/L, *p* = 0.005, data not shown). The most pronounced increase was observed for venom-specific IgG_4_, from 1.9 ± 0.7 mg_A_/L before VIT to 8.0 ± 2.0 mgA/L at day 28 after starting VIT (*p* < 0.001, Fig. 4c). When comparing changes of antibody levels, venom-specific IgE increased significantly more than total IgE, 4.1 ± 0.6-fold vs. 1.3 ± 0.1-fold (*p* < 0.001, Fig. 4d). The most pronounced change was observed for venom-specific IgG_4_, with a mean 12.8 ± 3.1-fold increase, which was significantly greater than the fold increase in venom-specific IgE (*p* = 0.004, Fig. 4d). Consistent with this, the venom-specific IgG_4_/IgE ratio increased from 0.28 ± 0.07 at baseline to 0.46 ± 0.07 at day 28 (*p* = 0.036, Fig. 4e).

## Discussion

Consistent with our hypothesis, early *Vespula* spp. VIT selectively reduced allergen-specific basophil activation, while non-specific basophil excitability remained unchanged. This was paralleled by an upregulation of Fc_ε_RI and Fc_γ_RIIb receptor numbers on basophils and an induction of venom-specific IgG_4_ serum levels.

Allergen-specific basophil activation requires effective cross-linking of Fc_ε_RI-bound IgE. Among other factors, this complex process is influenced by the overall level of allergen-specific IgE, the number of Fc_ε_RI on the cell surface, which determines the amount of receptor-bound IgE, the total allergen dose, and the number of epitopes per allergen [15, 16]. Recently, Puxkandl et al. reported an increase in the ratio of unoccupied to total Fc_ε_RI within the first hours after VIT induction [17]. We observed a dissociation between general basophil responsiveness and venom-specific excitability, suggesting that early VIT alters allergen-specific basophil activation whereas Fc_ε_RI-mediated signaling remains unchanged. Previous studies have described basophil desensitization during early VIT and suggested that this contributes to the rapid positive clinical effects observed after VIT initiation. Čelesnik et al. reported hyposensitization of Fc_ε_RI-activated basophils by day 5 of VIT [11], and a subsequent study showed reduced Fc_ε_RI -mediated CD63 basophil responses at this time [18]. Since impaired basophil responses might be expected to parallel with reduced Fc_ε_RI expression, the increase in Fc_ε_RI receptor numbers during VIT may appear paradoxical. However, Fc_ε_RI expression on basophils is dynamically regulated and influenced by IgE binding and cytokine-mediated priming. IL-3 has been shown to regulate basophil Fc_ε_RI expression and survival, while monomeric IgE may stabilize surface Fc_ε_RI [19]. Our data support the hypothesis that changes in receptor-bound allergen-specific IgE, rather than total Fc_ε_RI abundance alone, determine allergen-specific basophil activation. The concomitantly increased Fc_γ_RIIb mediates inhibitory signaling via co-aggregation with Fc_ε_RI [20]: IgG-allergen immune complexes crosslink Fc_γ_RIIb with Fc_ε_RI-bound IgE, thereby suppressing downstream Fc_ε_RI signaling and basophil degranulation [20, 21]. This inhibition is allergen-specific, which explains why anti-IgE- and fMLP-induced activation remained unchanged during early VIT. Component-resolved analyses have confirmed induction of IgG_4_ responses against single Hymenoptera venom allergens during VIT [22], consistent with our finding of an early venom-specific IgG_4_ response (approximately 13-fold induction). IgG_4_ may exert a blocking effect by competing with IgE for allergen binding [23] and, additionally, by forming immune complexes that engage Fc_γ_RIIb to initiate inhibitory signaling [21]. Since an increased surface Fc_γ_RIIb density lowers the threshold for inhibitory co-aggregation, the inhibitory effect of IgG_4_-mediated co-aggregation may be further amplified. Therefore, our data suggest that the reduction in allergen-specific basophil activation reflects the induction of venom-specific IgG_4_, which provides the humoral ligand for inhibitory Fc_γ_RIIb-Fc_ε_RI co-aggregation. This might be further amplified by concomitant Fc_γ_RIIb upregulation, which increases cellular sensitivity to this inhibitory signal. Our model implies that Fc receptors directly link the adaptive humoral response to basophil suppression during early VIT. Of note, the contribution of Fc_γ_RIIb-mediated signaling versus direct IgE blocking may vary in different allergens [24], and future experiments such as serum transfer assays and direct measurement of Fc_γ_RIIb occupancy are needed. Furthermore, our concept may help explain the temporal dynamics of VIT: initial clinical protection emerges within hours to days of high-dose allergen exposure during the up-dosing phase, driven by mast cells and basophil desensitization [10]. In addition, both the induction of IgG_4_ and the upregulation of Fc_γ_RIIb observed at day 28 may represent a second layer of protection. Long-term tolerance is subsequently consolidated by regulatory T-cell responses and sustained blocking antibody activity [10].

Our study has several limitations. First, due to the small size of our cohort, subgroup analyses according to severity of anaphylaxis, baseline IgE levels or clinical protection after re-sting during or after VIT were not feasible. Second, we did not extend the observation period beyond the first month of VIT; therefore, our data provide insight into early immune modulation but do not address long-term tolerance. Third, we did not directly measure Fc_γ_RIIb occupancy, IgG_4_-mediated co-aggregation, IgE-blocking activity, or the capacity of patient sera to inhibit venom-induced basophil activation in a transfer assay.

## Conclusions

Early *Vespula* spp. VIT selectively reduced venom-specific basophil activation, whereas anti-IgE- and fMLP-induced activation remained unchanged. This reduction became statistically significant by day 28, when it was accompanied by an induction of venom-specific IgG_4_ and increased Fc_γ_RIIb and Fc_ε_RI receptor numbers on basophils, distinct from the early, IgG_4_-independent desensitization established during up-dosing. Our data suggest that allergen-specific reduction of basophil activation is driven by two linked processes: Fc_γ_RIIb upregulation and increase of venom-specific IgG_4_ providing the humoral ligand for inhibitory signals, possibly by co-aggregation of Fc_γ_RIIb and Fc_ε_RI. Basophil excitability and Fc_γ_RIIb/Fc_ε_RI expression on basophils may play an important role within the complex mechanism of immune modulation during VIT. The relevance of Fc_γ_RIIb-Fc_ε_RI co-aggregation should be further investigated.

## Supplementary Information

Below is the link to the electronic supplementary material.

Supplementary Material 1

## Data Availability

No datasets were generated or analyzed during the current study.

## Abbreviations

BAT: Basophil activation test
fMLP: N-formylmethionyl-leucyl-phenylalanine
FITC: Fluorescein isothiocyanate
Fc_ε_RI: High-affinity immunoglobulin E receptor
Fc_γ_RIIb: Low-affinity immunoglobulin G receptor IIb
IDT: Intradermal testing
IL-3: Interleukin-3
MFI: Mean fluorescence intensity
PE: Phycoerythrin
SEM: Standard error of the mean
Ves v 5: *Vespula vulgaris* venom antigen 5
VIT: Venom immunotherapy
