# Supplementary material for: Vespula spp. venom immunotherapy: basophil excitability and FcεRI/FcγRIIb expression
Source: Allergy Asthma Clin Immunol. 2026 Sep 24;22:54. doi: 10.1186/s13223-026-01062-w (PMC13617696; doi:10.1186/s13223-026-01062-w)
Supplement: Supplementary file 1 [file 13223_2026_1062_MOESM1_ESM.docx]

**Supplementary Table 1:**

**Individual demographic and clinical patient characteristics.**

Demographic and clinical data, results of IDT, serum IgE levels, and BAT results prior to the start of VIT are shown for each of the 24 patients. Anaphylaxis grade was classified according to Ring and Messmer [14]. Atopy was defined by patient history of atopic dermatitis and/or asthma and/or allergic rhinoconjunctivitis. IDT threshold reflects the lowest venom concentration that elicited a positive skin reaction; "negative" indicates no positive reaction at any concentration tested. Total IgE and *Vespula* spp. venom- and honeybee venom-specific IgE were measured by ImmunoCAP™; manufacturer’s cut-off values for a positive result are given in square brackets. BAT results are shown as the percentage of CD63^+^ basophils after stimulation with 1000 or 100 ng/mL *Vespula* spp. venom, respectively.

#, patient number; d0, baseline (before start of VIT); f, female; IDT, intradermal test; IgE, immunoglobulin E; m, male; VIT, *Vespula* spp. venom immunotherapy.
